# Contribution of Cytoreductive Surgery and Hyperthermic Intraperitoneal Chemotherapy Videos on YouTube to the Learning Curve in COVID-19 Pandemic Process

**DOI:** 10.7759/cureus.16820

**Published:** 2021-08-02

**Authors:** Şevket Barış Morkavuk, Atilla Badem

**Affiliations:** 1 Surgical Oncology, Ankara City Hospital, Ankara, TUR; 2 General Surgery, Ankara City Hospital, Ankara, TUR

**Keywords:** cytoreductive surgery, hyperthermic intraperitoneal chemotherapy, surgical education, youtube, peritoneal carcinomatosis, learning curve

## Abstract

Introduction: YouTube is a free video-sharing platform, which is watched by residents or specialists in order to catch up on their skills, see new techniques, and have information. In this study, our purpose is to evaluate whether or not these videos are an appropriate educational source for surgeons by analyzing their quality and content, in an environment of reduced elective operations due to the COVID-19 pandemic.

Methods: YouTube video search was performed by entering keywords such as CRS and HIPEC, cytoreductive surgery and hyperthermic intraperitoneal chemotherapy, cytoreductive surgery, and HIPEC. A total of 115 videos were found. Videos were divided into two groups as those containing operation videos and those that do not (slides, congress presentations, and informative videos). And then, for evaluating operation videos, a scoring system was defined based on CRS + HIPEC steps as defined by Sugarbaker and video quality.

Results: There were 45 operation videos and 70 videos of other content. The total number of views, number of views per day, and the total number of likes and comments were all significantly higher in the operation video group (p values 0.003-0.002-0.004-0.002). No statistically significant difference was found in the median values of the number of views, likes, and dislikes; the number of views per day; the number of comments; and the dates of uploads within different video-scoring system groups.

Conclusion: Our study shows that there is an increasing interest in CRS + HIPEC videos on YouTube. On the other hand, when the educational value of these videos that are receiving attention is considered, we believe it is not right to adjudicate due to the fact that the numbers are very low.

## Introduction

General surgery residency is a long and wearing process. Varying among countries, mean labor hours are between 48 and 80 hours a week [1,2]. In Turkey, standard daily work hours of 40 hours a week plus 56 hours of night shifts exceeds 96 hours a week for general surgery residents. Despite challenging working hours, training of general surgery residents may still be insufficient in certain types of operations. In the general surgery residency curriculum of our country, sufficient interventional levels are defined for a resident to become a general surgeon. The first level is to have knowledge on how the procedure is performed. The second level is to have the ability to perform the operation under supervision. The third level is to be able to perform common non-complicated procedures. The fourth level is being able to do every kind of the procedure, whether or not it is complicated. The education level of cancer surgery is between 2 and 3 [3]. In our country, these levels are similar to the rates of the United Kingdom Intercollegiate Surgical Curriculum Program before the revision in 2018 [4]. Many nonmalignant elective and emergent surgeries show level 4 ability. Also, during the resident training process, the amount of cases that should be operated under the supervision of a mentor is 400. However, due to the lack of a standard level between education and training foundations, many residents complete their training without having sufficient education. As a result, surgeons keep away from cases that they feel insufficient about, in order to not to face medicolegal problems, rather prefer performing cases that they feel more confident. At this exact point, the importance of continuing medical education comes up. Within this context, surgeons seek a way to complement the lack of education after the residency. Either by joining varying courses or by video-training sets and video platform sites, they try to support themselves visually. One of these sites is YouTube, which we all know too well.

YouTube is a free video-sharing platform visited by 95% of internet users actively, which started broadcasts in 2005 [5]. YouTube has over two billion users, and its monthly visitor count is up to 8.6 billion for 2019. One billion videos are watched every day, and 500 hours of new videos are uploaded every minute [6]. Undoubtedly, videos containing the health sector and surgical procedures have an important place among them. Residents or specialists watch YouTube videos in order to catch up on their skills, see new techniques, and have information. Studies showed that especially laparoscopic surgery videos are being watched, and these videos provide training on the use of laparoscopic instruments. There is also the fact that visual multimedia use increases the ability to learn [7].

In this case, could YouTube be a potential training platform for CRS + HIPEC (cytoreductive surgery + hyperthermic intraperitoneal chemotherapy) operation, which has high morbidity and mortality rates and long operation times? Peritoneal carcinomatosis is a condition that shows poor prognosis and low survival expectation, which may appear at many end-stage cancers. CRS was defined in 1930, followed by hyperthermic chemotherapy in 1977 [8]. The peritonectomy with CRS and HIPEC of today was standardized by Sugarbaker in 1995 [9]. CRS + HIPEC shows positive survival rates along with its success in managing peritoneal disease in chosen patients (patients with optimal peritoneal cancer index). However, it is a very aggressive procedure with 1%-5% mortality and 10%-30% morbidity [10]. At first, it did not have the expected popularity among surgeons due to these high morbidity and mortality rates. Lately, with increasing surgical oncology, gynecologic oncology, and gastroenterology clinics, there has been an increase in CRS + HIPEC operations. It is challenging to define a certain number for this increase. In the United States alone, 16,58,370 patients were diagnosed with cancer in 2005 of which 29,000 to 41,000 patients were believed to be appropriate for CRS + HIPEC procedure [11,12]. However, this increase in operations did not have a reflection on resident training sufficiently. This may be due to long operation length, high morbidity and mortality, cases being operated by specialist surgeons (due to legal obligations), gradual learning curve of the operation, low operation numbers when compared to resident numbers, etc.

In this study, we investigated the CRS + HIPEC videos uploaded to the YouTube platform. Our purpose is to evaluate whether or not these videos are an appropriate educational source for surgeons by analyzing their quality and content in an environment of reduced elective operations due to the COVID-19 pandemic.

## Materials and methods

On October 14, 2020, a YouTube video search was performed by entering keywords CRS and HIPEC, cytoreductive surgery and hyperthermic intraperitoneal chemotherapy, cytoreductive surgery, and HIPEC. A total of 61 videos were found in CRS + HIPEC search, among which 71 videos were in cytoreductive surgery and HIPEC search and 57 were in hyperthermic intraperitoneal chemotherapy. Shared videos in each group were found in order to avoid repeats. A total of 118 videos were found. By taking the number of views into consideration, videos were sorted by view counts. Due to the possibility that new videos of the subject may be uploaded, our list of videos was saved as a playlist. This way prevented adding more videos to the study during the evaluation of the videos and statistical analysis.

Videos were watched together by a surgical oncologist and a general surgery resident. Each video was evaluated for video quality, verbal expression, video language, length, step-by-step definition of the operation, the number of views and likes, subtitle availability, duration since the day of upload, and the number of comments. Comments of a surgical oncologist who knows the CRS + HIPEC procedure steps and a general surgery resident who is still on the learning curve and does not have sufficient knowledge of the procedure were noted. First of all, videos were divided into two groups as those containing operation videos and those that do not (slides, congress presentations, and informative videos). And then, due to the fact that there is no standard method for evaluating operation videos, a scoring system was defined based on CRS + HIPEC steps as defined by Sugarbaker [9] and video quality (Table 1). There are 10 items in this scoring system in total. Each item may provide 0 or 1 point. Videos were evaluated on these 10 items and a final scoring was made with a consensus between 0 and 10 points. Video scores were categorized into three groups: poor (0-3), moderate (4-7), or good (8-10) just as in other similar studies.

**Table 1:** Video-scoring system HIPEC, Hyperthermic intraperitoneal chemotherapy.

Item No.	Category	Assessment	Score
1	Voice narration	Absent	0
		Present	1
2	Subtitle narration	Absent	0
		Present	1
3	Video resolution quality	Low	0
		High	1
4	Patient information	Absent	0
		Present	1
5	Operation plan	Absent	0
		Present	1
6	PCI calculating	Absent	0
		Present	1
7	Peritonectomy and organ resection demonstration	Absent	0
		Present	1
8	HIPEC machine and technical description	Absent	0
		Present	1
9	Drug information	Absent	0
		Present	1
10	Perioperative information	Absent	0
		Present	1
Total Score			10
˟ Total score: 0-3, poor; 4-7, moderate; 8-10, good.			

Institutional Review Board and Ethical approval were not received for the present study as only the common data open to public access were used.

Statistics

Data were analyzed using Statistical Package for the Social Sciences (SPSS) version 22.00 (IBM Inc., Armonk, USA). Pearson chi-square and Fisher tests were used for evaluation of nominal data of each group, student’s T-test for scale parametric data, and Mann-Whitney U test for scale nonparametric data. One-way ANOVA (analysis of variance), Kruskal-Wallis test, and posthoc multiple comparison (Bonferroni) test were used for the analysis of multiple groups. P < 0.05 was deemed statistically significant.

## Results

A total of 118 videos were found containing keywords CRS + HIPEC, cytoreductive surgery and HIPEC, hyperthermic intraperitoneal chemotherapy in the YouTube search engine. Two videos were ruled out due to having a patient interview and one for containing only a pathology specimen. A total of 115 videos were evaluated; 39.1% of the videos included in this study (n = 45) consisted of operation videos, 27.8% were informative interview videos (n = 32), 10.4% were made of animation videos (n = 12), and 22.6% were presentations of congresses; 73.9% of the videos had voice narrative (n = 85), and 85.8% of them were in English. Only 59.1% of the videos had subtitles available (n = 68), and all of them were in English. The median number of views was 8177.70 (range 9-127637) and views per day were 7.98 (range 0.03-290). The distribution of the videos in the study according to their characteristics was shown in Table 2.

**Table 2:** Distribution of the videos in the study according to characteristics SD, Standard deviation.

Video Types: n(%)	
Operation videos	45 (39.1%)
Animation videos	12 (10.4%)
Congress presentation videos	26 (22.6%)
İnformative interview videos	32 (27.8%)
Voice Narration: n(%)	
Absent	30 (26.1%)
Present	85 (73.9%)
Subtitle Narration: n(%)	
Absent	47 (40.9%)
Present	68 (59.1%)
Video Narration Language: n(%)	
Absent	30 (26.1%)
English	73 (63.5%)
Others	12 (10.4%)
Comment: n(%)	
Absent	71 (61.7%)
Present	44 (38.3%)
Video resolution quality: n(%)	
Low	31 (26.9%)
High	84 (73.1%)
Total view, number, mean ± SD, range	8177.70 ± 20470.68 (9-127637)
Views per day, number, mean ± SD, range	7.98 ± 29.70 (0.03-290)
Time since upload, day, mean ± SD, range	1344.30 ± 1015.44 (4-3918)
Video length, second, mean ± SD, range	931.37 ± 987.12 (82-7058)
Comment, number, mean ± SD, range	2.82 ± 8.74 (0-77)
Like, number, mean ± SD, range	27.52 ± 63.73 (0-410)
Dislike, number, mean ± SD, range	1.94 ± 4.92 (0-27)

Videos were divided into two groups as those containing operation videos and those that do not (slides, congress presentations, and informative videos). As to this setup, there were 45 operation videos and 70 videos of other content. Subsequently, the characteristics of each group were statistically compared. When the voice narration of the videos was compared, there was a statistically significant difference between the operation video group and the other group of nonoperational videos (p < 0.01). Both variables were higher in the group of nonoperational videos. However, when comments were evaluated, the operation video group had more comment rate, whereas 50 videos of the other group had no comments at all (p = 0.008). Due to the fact that none of the scale variables could pass the homogeneity and distribution test, It was evaluated with Mann-Whitney U test. The total number of views, number of views per day, and the total number of likes and comments were all significantly higher in the operation video group (p values 0.003-0.002-0.004-0.002, respectively). Median values of dislikes and video lengths showed no statistically significant difference (Table 3).

**Table 3:** Analysis of video characteristics by video type groups

Characteristics	No. of Videos (%)		p value
	Operation Videos	Other Videos	
	(45 videos, 39.1%)	(70 videos, 60.9%)	
Voice Narration: n(%)			p < 0.01
Absent	25 (21.73%)	5 (4.34%)	
Present	20 (17.39%)	65 (56.52%)	
Subtitle Narration: n(%)			p < 0.01
Absent	28 (24.34%)	19 (16.52%)	
Present	17 (14.78%)	51 (44.34%)	
Video Narration Language: n(%)			p < 0.01
Absent	25 (21.73%)	5 (4.34%)	
English	19 (16.52%)	54 (46.95%)	
Others	1 (0.86%)	11 (9.56%)	
Comment: n(%)			p = 0.008
Absent	21 (18.26%)	50 (43.47%)	
Present	24 (20.86%)	44 (38.26%)	
Video Resolution Quality: n(%)			p = 0.003
Low	19 (16.52%)	12 (10.43%)	
High	26 (22.60%)	58 (50.43%)	
Total views, number, median, range	1550 (50-127637)	451.5 (9-95403)	p = 0.003
Views per day, number, median, range	1.78 (0.09-290)	0.60 (0.03-54.54)	p = 0.002
Time since upload, day, median, range	1337 (4-3918)	1029.5 (27-3918)	p = 0.422
Video length, second, median, range	597 (84-7058)	524 (82-3655)	p = 0.347
Comment, number, median, range	1 (0-77)	0 (0-28)	p = 0.002
Like, number, median, range	10 (0-221)	2.5 (0-410)	p = 0.004
Dislike, number, median, range	0 (0-27)	0 (0-25)	p = 0.110

All the videos were divided into two groups based on median values of the number of views per day (median value 0.86). There were 58 videos in the group below the median value and 57 in the other group above. Statistical analysis of both groups was performed. As a result, between the groups of the different numbers of views, there was no statistically significant difference in video duration, presence of voice narrative, video language, and subtitle availability. However, there was a significant difference between the groups when compared for video content and video resolution. Twenty-nine out of 45 operation videos were in the group above median value, whereas 42 out of 70 videos of other contents were in the group that was below (p = 0.011).

Table 4 shows statistical results between groups referred to as poor, moderate, and good, which were categorized in line with the video-scoring system. The poor-grade group included 13, the moderate group included 23, and the good-grade group included nine videos. There was no statistically significant difference between groups of different video scores on video resolution, subtitle availability, and the number of comments. On the other hand, a significant difference was found in voice narrative and English language categorization between the groups. According to this, 13 of 20 videos that have voice narrative were in the moderate group, and 13 of the 19 videos that have the English language were also in the moderate group (p = 0.044/p = 0.034). The only homogeneous distribution in scale variables was the points of the video-scoring system. No statistically significant difference was found in the median values of the number of views, likes, and dislikes; the number of views per day; the number of comments; and the dates of uploads within different groups. A statistically significant difference was found in the scores of three different video quality groups (p < 0.01). This difference was based on the relation between poor-moderate, poor-high, and moderate-high groups according to the posthoc multiple comparison (Bonferroni) test.

**Table 4:** Analysis of video characteristics by video quality groups ˟Kruskal-Wallis test ˟˟One-way ANOVA test ANOVA, Analysis of variance.

Characteristics	Video quality groups (n%)			p value
	Poor (13, 28.9%)	Moderate (23, 51.1%)	Good (9, 20%)	
Voice Narration: n(%)				0.044˟
Absent	11	10	4	
Present	2	13	5	
Subtitle Narration: n(%)				0.116˟
Absent	11	13	4	
Present	2	10	17	
Video Narration Language: n(%)				0.034˟
Absent	11	10	4	
English	1	13	5	
Others	1	0	0	
Comment: n(%)				0.388˟
Absent	5	13	3	
Present	8	10	6	
Video Resolution Quality: n(%)				0.078˟
Low	8	6	5	
High	5	17	4	
Total View, number, median, range	2025 (78-127637)	1020 (50-55324)	11586 (129-48602)	0.382˟
Views per day, number, median, range	0.94 (02-55,63)	1.73 (0,09-290)	6.65 (0,1-27,91)	0.978˟
Time since upload, day, median, range	1705 (327-3157)	822 (4-3918)	1537 (1254-1741)	0.071˟
Video length, second, median, range	561 (84-7058)	591 (180-4593)	1616 (410-1620)	0.296˟
Comment, number, median, range	2 (0-77)	0 (0-15)	6 (0-33)	0.147˟
Like, number, median, range	11 (0-214)	7 (0-176)	45 (0-221)	0.478˟
Dislike, number, median, range	1 (0-27)	0 (0-12)	2 (0-17)	0.325˟
Group score, number, mean ± SD, median; min-max	1.92 ± 0.95 2; (1-3)	5.39 ± 0.98 5; (4-7)	9.22 ± 0.97 10; (8-10)	150,288F <0.01˟˟

## Discussion

In most professions in which hand skills are important and side instruments are used, it is possible to get ready for professional work-life using simulators or various training sets. However in the health sector, especially in the surgical field, these possibilities are limited due to the fact that working material is a human life. Lately, depending on the improvements on minimal invasive surgery, training sets or animal laboratories may be used for learning surgical techniques and use of surgical tools. However, these possibilities are not available for most of the surgical procedures, and most of the health personnel cannot acquire them. Therefore, surgery residents and specialists watch operational videos on YouTube and similar video platform sites. Also, some surgical societies suggest that the YouTube video platform may be a follow-up for surgery resident training. International Endohernia Society 2011 and 2015 Guide and European Hernia Society 2009 Guide tell that YouTube contributes surgeons a lot for totally extraperitoneal (TEP) procedure [13,14].

In the literature, there were no studies evaluating CRS + HIPEC videos in YouTube. On the other hand, we found many studies concerning laparoscopic bariatric surgery, laparoscopic inguinal hernia repair, laparoscopic cholecystectomy, and laparoscopic and robotic prostatectomy in YouTube. In most of these studies, authors included the first 120 videos when evaluating YouTube videos because in a study survey among internet users, it was found that most of the participants only investigated the first three pages [15]. However, in our study, CRS + HIPEC keyword search showed a total of 118 results. We believe this is due to the fact that the CRS + HIPEC procedure can only be performed in chosen high-end health centers; surgeons who chose to operate peritoneal surface diseases are a few; this procedure has lower incidence than others or that operation length is long.

In this study, only 45 (38.13%) of 118 videos that are included were made of operation videos. The exclusion rate was 2.54%. In a study by Yağız et al., videos containing operation videos were 82.5%, and the exclusion rate was 54.16% [16]. Similarly, in another study by Lee et al., these rates were 73% vs 27%, respectively [17]. The advantage of these studies was that the procedures they investigated were highly popular (laparoscopic inguinal hernia repair and laparoscopic cholecystectomy), and therefore YouTube had many examples of them.

A standard CRC + HIPEC procedure is six to seven hours long depending on the inclusion of peritonectomy and multiple organ resections. In a study that included 370 cases by Polanco et al., this duration was reported as 430 minutes [18]. No doubt, a video this long is not expected to be uploaded to YouTube. In our study, the mean video length was 15.51 minutes and the longest one was 117.63 minutes. When this mean length was compared to the actual operational duration in a study by Polanco et al., a ratio as low as 0.03 comes up. In order to evaluate this ratio, we investigated the studies about YouTube and surgical videos. Because of the fact that there is no other study on CRC + HIPEC and YouTube in literature, we compared this ratio with studies of other surgical videos. In a study of laparoscopic total extraperitoneal hernia by Bhandarkar et al., [19] the actual operation duration was 54 minutes, and in another study on YouTube videos by Yağız et al., the median video length was 16.25 [16]. Their ratio to each other is 0.30. In order to get this ratio for CRS + HIPEC videos, videos should be 120 minutes, approximately. Therefore, CRS + HIPEC videos on YouTube are very short.

Celentano et al. reported that 86.7% of surgery residents watch video platform sites of which 98% are YouTube [20]. According to this study, residents’ video preferences are operational videos, surgical instruments, preoperative-perioperative-postoperative information. Despite the popularity of YouTube, there is no standard method for the evaluation of medical videos. In 2018, LAP-VEGaS (Laparoscopic Surgery Video Educational Guidelines) was published for laparoscopic surgery videos [21]. Most of the studies on laparoscopic surgery and YouTube are performed according to this guideline. Videos were categorized into poor, moderate, and good quality and analyzed in between. However, there is no standard categorization or a guideline for open surgery videos. The biggest limitation of this study was on this step. There is no standard categorization method for open surgery as well as there is no proven scale for evaluating CRS + HIPEC video quality. Thus, we developed a scoring system by evaluating the visual and audio contents of the videos by the light of recent literature and the steps of the procedure as standardized by Sugarbaker [9]. Accordingly, operation videos were grouped as poor, moderate, and good, and statistical analyses were performed for each group. In the study of laparoscopic hernia repair and YouTube by Yağız et al., operation videos were divided into three categorizations: good, moderate, and poor. The good-grade group showed a significant difference in likes and comment numbers [16]. Similar to our study, there was no significant difference in views per day or the total number of views. Also in another study of laparoscopic cholecystectomy and YouTube by Lee et al., groups showed no difference in the number of views, comments, and likes [17]. However, we found a significant difference when we compared operation videos with other videos, which was the first step of this study. The total number of views, views per day, and the number of comments and likes were significantly higher in the group of operation videos. We came to the conclusion that CRS + HIPEC operation videos draw attention and were watched on YouTube.

One of the main reasons behind the increase in the number of views of surgical videos on YouTube is the learning curve. The term ‘learning curve’ was first defined by TP Wright in 1936 [22]. It is defined as the reduction of the time needed for the production of one unit when the cumulative production rate doubles. Every surgical operation has a different learning curve. CRS + HIPEC is a procedure that needs more cases than others for the steeping process. Kusamura et al. evaluated the learning curves reported by two CRS + HIPEC centers in Italy. In this study, they reported that 137 cases were needed in order to reduce the high morbidity and insufficient cytoreduction rates in the center of Milan, while this number was found to be 126 in the center of Bentivoglio [23]. In a multiple-centered metaanalysis by Rajeev et al., the number of cases needed for the learning curve of CRS + HIPEC procedure was reported to be 55 (range 24-123) [12]. These numbers require too much time to get. For this reason, the place of YouTube or other video-sharing sites in continuing medical education cannot be ignored.

## Conclusions

In the popular video platform, YouTube, CRS + HiPEC operation videos draw attention even though they are low in numbers. This shows the interest in CRS + HiPEC operation videos. On the other hand, when the educational value of these videos that are receiving the attention is considered, we believe it is not right to adjudicate due to the fact that the numbers are very low. This handicap could be removed by uploading the videos containing operations of related societies and by professors who have the required information and experience on peritoneal surface malignancy to YouTube. Besides, these uploads may pave the way for new CRS + HiPEC studies associated with YouTube.

## Appendices

Tablolar Efsaneler

Tablo 1. Video Puanlama Sistemi

Tablo 2. Çalışmadaki Videoların Özelliklerine Göre Dağılımı

Tablo 3. Video türü gruplarına göre video özelliklerinin analizi

Tablo 4. Video kalite gruplarına göre video özelliklerinin analizi

beyannameler

Kısaltmalar

KRS: sitoredüktif cerrahi; HiPEC: hipertermik intraperitoneal kemoterapi.

Kurumsal inceleme kurulu beyanı: Kamu erişimine açık ortak veriler kullanıldığından bu çalışma için Kurumsal İnceleme Kurulu ve Etik onayı alınmamıştır.

rekabet eden çıkarlar

İsimleri hemen aşağıda listelenen yazarlar, bu yazıda tartışılan konu veya materyallerde herhangi bir finansal çıkar veya finansal olmayan çıkarı olan herhangi bir kuruluş veya kuruluşla bağlantısı veya ilgisi olmadığını onaylar. Makale başlığı: Covid-19 pandemi sürecinde Youtube'daki sitoredüktif cerrahi (CRS) ve hipertermik intraperitoneal kemoterapi (HIPEC) videolarının öğrenme eğrisine katkısı.

Yazarın ismi: Şevket Barış Morkavuk, Atilla Badem

Finansman

Finansman ve diğer destek yok

Veri ve materyallerin mevcudiyeti

Mevcut çalışma sırasında kullanılan ve/veya analiz edilen veri setleri, makul talep üzerine ilgili yazardan temin edilebilir.

Yazar katkıları

Şevket Barış Morkavuk: Makale düzenleme, İstatistiksel analiz ve hazırlama

Atilla Badem: Verilerin ve algoritmaların kalite kontrolü
